# A novel material of cross-linked styrylpyridinium salt intercalated montmorillonite for drug delivery

**DOI:** 10.1186/1556-276X-9-378

**Published:** 2014-08-03

**Authors:** Jing Cui, Qingqing Wang, Xiaodong Chen, Qufu Wei

**Affiliations:** 1 Key Laboratory of Eco-textiles, Jiangnan University, Wuxi 214122, China

**Keywords:** Montmorillonite, Intercalation, SbQ

## Abstract

A facile synthesis of a styrylpyridinium salt (SbQ)/montmorillonite (MMT) via cationic exchange interactions between styrylpyridinium species (specifically SbQ) and MMT platelets is reported in this work. The SbQ-MMT solutions were irradiated under ultraviolet (UV) light for a specific time to obtain the cross-linked SbQ-MMT materials. Scanning electron microscopy and atomic force microscopy analyses revealed the structures and morphologies of MMT and modified MMT. X-ray diffraction and transmission electron microscope analyses indicated that the basal spacing increased from 1.24 to 1.53 nm compared with the pristine MMT, which proved that SbQ had interacted with MMT. Thermal gravimetric analysis curves showed that the amount of SbQ in the MMT interlayers was 35.71 meq/100 g. Fourier transform infrared spectroscopy also confirmed the intercalation of SbQ species into MMT interlayers, and UV spectroscopy was used to follow up the cross-linking of SbQ-MMT. This novel material has potential applications in drug delivery, and it can also be used as an additive to improve the mechanical properties of polymers.

## Background

SbQ (a styrylpyridinium salt), similar to surfactants, is an amphiphilic sensitizer of the styrylpyridinium family [1], and it produces a very planar stacked rod-like micelle structure. Such a structure makes it possible to stack the molecules with the hydrophobic regions one above the other, with the aldehyde and nitrogen-methyl groups alternating, and finally produces an aggregate [2]. SbQ can react with amino groups of proteins to improve the protein stabilization [3]. Moreover, it can be dimerized via the [2 + 2]-cycloaddition reaction under ultraviolet (UV) irradiation [4]. According to Tao et al. [5], cross-linking of the hydrophobic core via dimerization reaction of the SbQ molecules induced by UV light ultimately produced cross-linked micelles because of hydrophobic interactions between SbQ molecules. Hence, the cross-linked SbQ-montmorillonite (MMT) has potential applications for hydrophobic drug delivery and can be used as an additive into polymeric composites and improve the stability and mechanical properties of polymers [6-9].

In recent years, modified MMTs have been extensively used as drug delivery carriers and for protein adsorption [10-13]. Banik et al. introduced soy flour (SF)-MMT nanoparticles cross-linked with glutaraldehyde (GA) as a carrier for isoniazid [10]. Joshi et al. investigated the intercalation of timolol maleate (TM) into MMT as a sustained drug carrier [11]. Sarıoğlan et al. studied the cationic pigment-intercalated MMT as the latent print development powder [12]. Madurai et al. found an intestine-selective drug delivery system via the intercalation of captopril (CP) into the interlayers of MMT [13]. MMT is one of the smectite group having two silica tetrahedral sheets layered between an alumia octahedral sheet. In nature, the charge imbalance in the structure is neutralized by adsorption of sodium or calcium ions in the interlayer, which makes intercalation possible by cation exchange with metallic/organic cations [12]. MMT has attracted a great deal of attention in recent years for drug delivery applications due to its good physical and chemical properties [10].

In this work, a styrylpyridinium salt and MMT was used to prepare SbQ-MMT cross-linked hybrid materials by UV light irradiation. Since organic-inorganic hybrids prepared by the intercalation of organic species into layered inorganic solids contain properties of both the inorganic host and the organic guest in a single material, it is a useful and convenient route to prepare SbQ-MMT hybrids [11]. The preparation process involved the following two steps: firstly, the cation of SbQ was exchanged with the sodium of MMT and the SbQ was intercalated into the interlayers of MMT. Secondly, the SbQ-MMT solution was irradiated under UV light to get the cross-linked hybrid materials. There were hydrophobic interactions between SbQ molecules via UV cross-linking [1]. The aldehyde (−CHO) group of SbQ has a potential to interact with − NH_2_ groups of proteins and this interaction could be used for drug delivery applications. More importantly, after UV light irradiation, the cross-linked SbQ may have potential applications such as hydrophobic drug delivery [5], stimuli-responsive field [14,15], and passivation layer [16].

## Main text

### Experimental

#### Materials

1-Methyl-4-[2-(4-formylphenyl)-ethenyl]-pyridiniummethosulphate (SbQ) was purchased from Shanghai Guangyi Printing Equipment Technology Co. Ltd (Shanghai, China). Sodium montmorillonite (Na-MMT) was a kind gift from Zhejiang Fenghong Chemical Co. Ltd. (Huzhou, Zhejiang, China; the cation exchange capacity of the sodium MMT was 92 meq/100 g). Deionized water was used for the preparation of all solutions.

#### Synthesis of cross-linked SbQ-modified MMT

**Figure 1:**
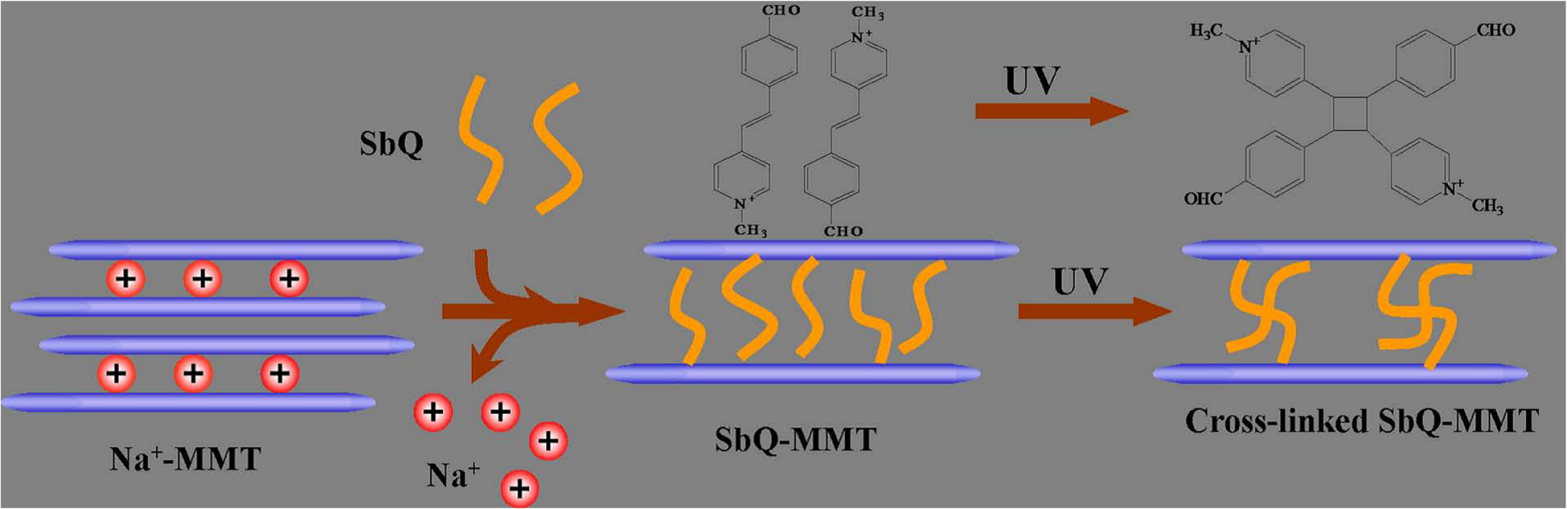
Illustration of the synthesis procedure of cross-linked SbQ-MMT materials.

SbQ-modified MMT (SbQ-MMT) was prepared by cation exchange between Na^+^ in MMT galleries and SbQ cations in aqueous solution according to a modified literature method. Na-MMT (1 g) dispersed in 50 mL of deionized water was vigorously stirred for 3 h [17]. An aqueous solution (50 mL) containing SbQ (1 g) was added under stirring for 3 h to obtain SbQ-MMT. To obtain cross-linked particles, the SbQ-MMT solutions were irradiated under a PowerArc UV100 Lamp (UVPS, Chicago, IL, USA) for a specific time. The sample was separated from the solution by vacuum filtration, and then washed repeatedly with deionized water, followed by drying under a vacuum for 12 h at 60°C. The synthesis method as described above is illustrated in Figure 1.

### Characterizations

The shapes and surface morphologies of the samples were investigated by atomic force microscopy (AFM, Benyuan CSPM 4000, Shenzhen, China) with tapping mode under aqueous media and scanning electron microscopy (SEM, Hitachi SU1510; Hitachi Ltd., Beijing, China). To determine the particle size and size distribution, the AFM images were analyzed using the image analyzer software. XRD scans of the MMT and dried SbQ-MMT powder were obtained by X-ray diffraction patterns (XRD, MAC Science Co. Ltd. MXP 18 AHF, Yokohama, Japan) with Cu-Kα radiation and the results were confirmed by a transmission electron microscope (TEM, JEOL2010, Akishima-shi, Japan; Philips, Amsterdam, Netherlands). The intercalation of SbQ molecules in Na-MMT layers after cation exchange and UV irradiation were also examined by Fourier transform infrared spectroscopy (FTIR, Nicolet Nexus, Thermo Electron Corporation, Waltham, MA, USA) in the range 4,000 to 500 cm^−1^, using KBr-pressed method. The cross-linking of SbQ was followed by UV-vis spectroscopy.

The amount of SbQ intercalated in MMT was conducted by thermal gravimetric analysis (TGA, TGA/SDTA851e) at a heating rate of 10°C/min in a nitrogen flow.

## Discussion

### Morphology analysis

**Figure 2:**
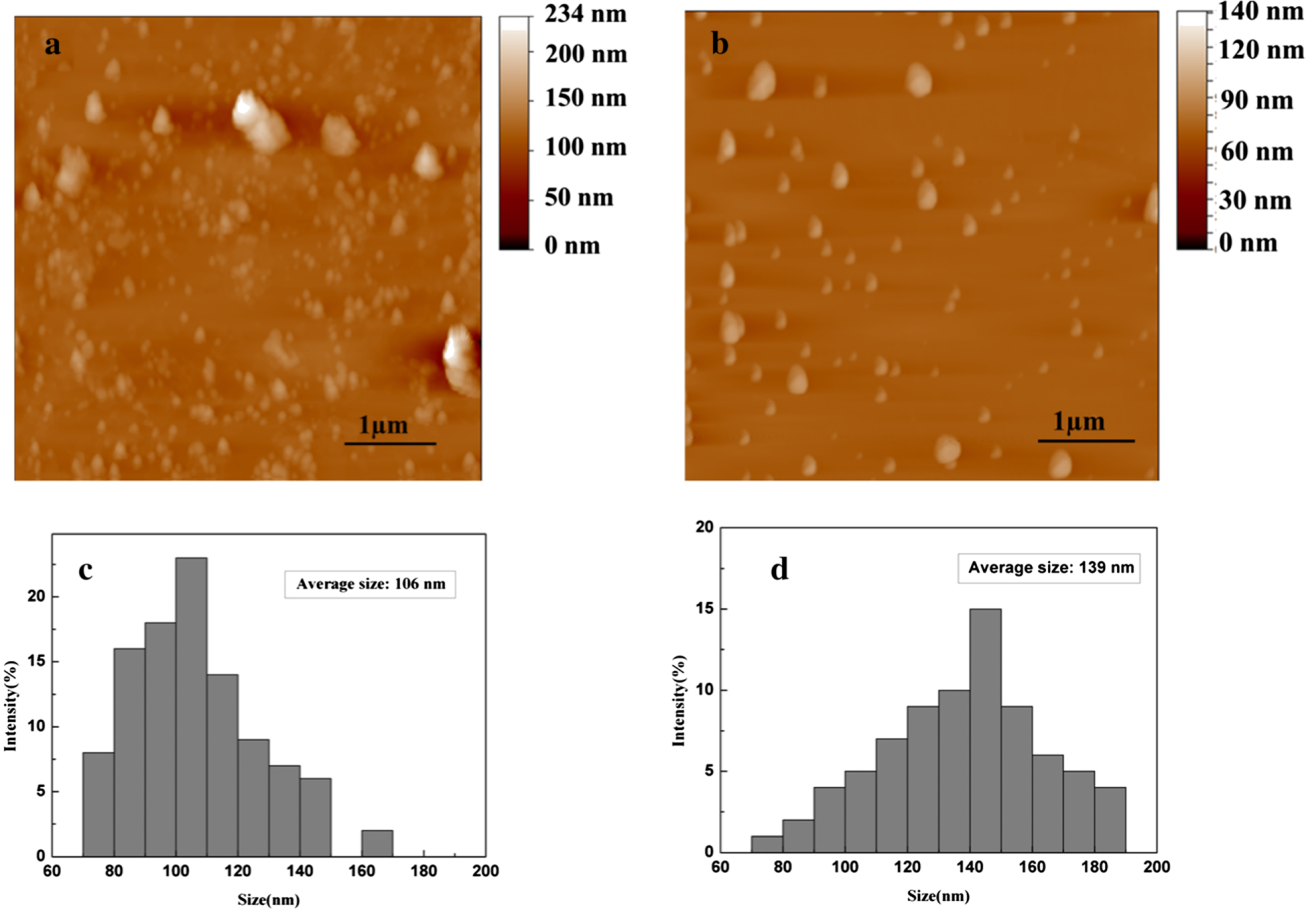
**AFM images and size distribution. (a) (c)** MMT. **(b) (d)** SbQ-MMT. **(c)** SD = 20.2; **(d)** SD = 45.

**Figure 3:**
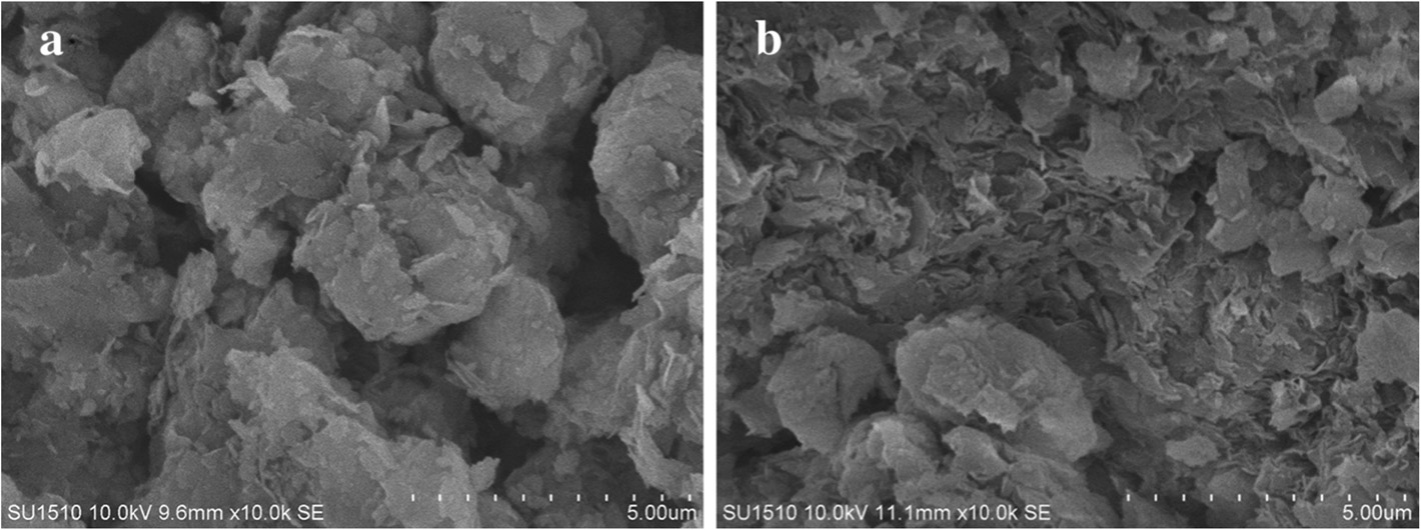
**SEM images. (a)** MMT. **(b)** SbQ-MMT.

AFM images were obtained to visualize the shapes and surface morphologies of MMT and cross-linked SbQ-MMT in aqueous solution, as presented in Figure 2. It was observed that the morphology of MMT was heterogeneous due to the molecular aggregation in the solution in Figure 2a. Cross-linked SbQ-MMT showed a spherical morphology which probably resulted from the presence of hydrophobic interactions among the SbQ molecules and the presence of excess negative charges on the chain in Figure 2b. Average particle size and size distribution of MMT and cross-linked SbQ-MMT in aqueous solution were also measured. From the bar graphs as presented in Figure 2c,d, it could be observed that the average particle size of MMT was less than SbQ-MMT. The average particle sizes of MMT and SbQ-MMT were 80 to 120 nm and 100 to 180 nm, respectively. The increase in particle size indicated that SbQ had been intercalated into MMT. Size increase due to aggregation of the hydrophobic SbQ-MMT particles in the aqueous environment also cannot be ignored.Figure 3 compares the morphology of MMT and cross-linked SbQ-MMT powder. As shown in Figure 3a, it could be found that MMT with layered structure aggregated into large particles. Compared with pristine MMT, the partially exfoliated MMT/SbQ composites could be clearly seen in Figure 3b. Cross-linked SbQ-MMT showed a multi-layer structure, and some exfoliated layers were also observed which further confirmed that the interlayer structure had been changed by the existence of SbQ.

**Figure 4:**
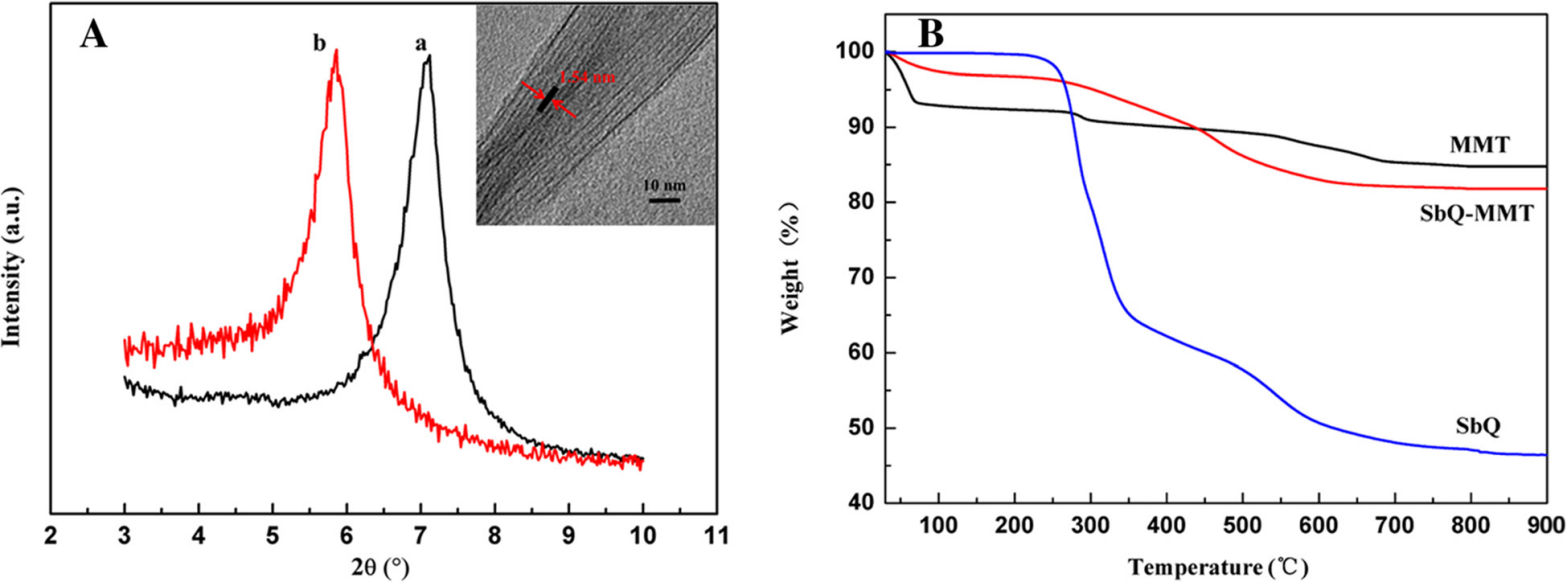
**XRD patterns and TEM image and TGA curves. (A)** XRD patterns and TEM image: (a) MMT, (b) SbQ-MMT, and TEM (inset) of SbQ-MMT. **(B)** TGA curves.

More detailed evidences are shown in Figure 4A. The pristine MMT showed a typical XRD pattern with the basal spacing of 1.24 nm and intercalation of SbQ led to a significant increase in interlayer spacing and a decrease in 2θ. The increased basal spacing indicated that SbQ had been effectively intercalated into the interlayers of MMT. It could also be seen from the TEM image (inset) that the MMT was comprised of many parallel silicate layers with about 1.5 to 2 nm interlayer spacing. The interlayer spacing was much larger than the original 1.24 nm of MMT, which gave direct evidence that the SbQ molecules had been intercalated into MMT. From the TGA curves (Figure 4B), the amount of SbQ in the MMT interlayers was about 7.57% (35.71 meq/100 g) [12], which is less than the cation exchange capacity of the sodium MMT.

### Structural analysis

**Figure 5:**
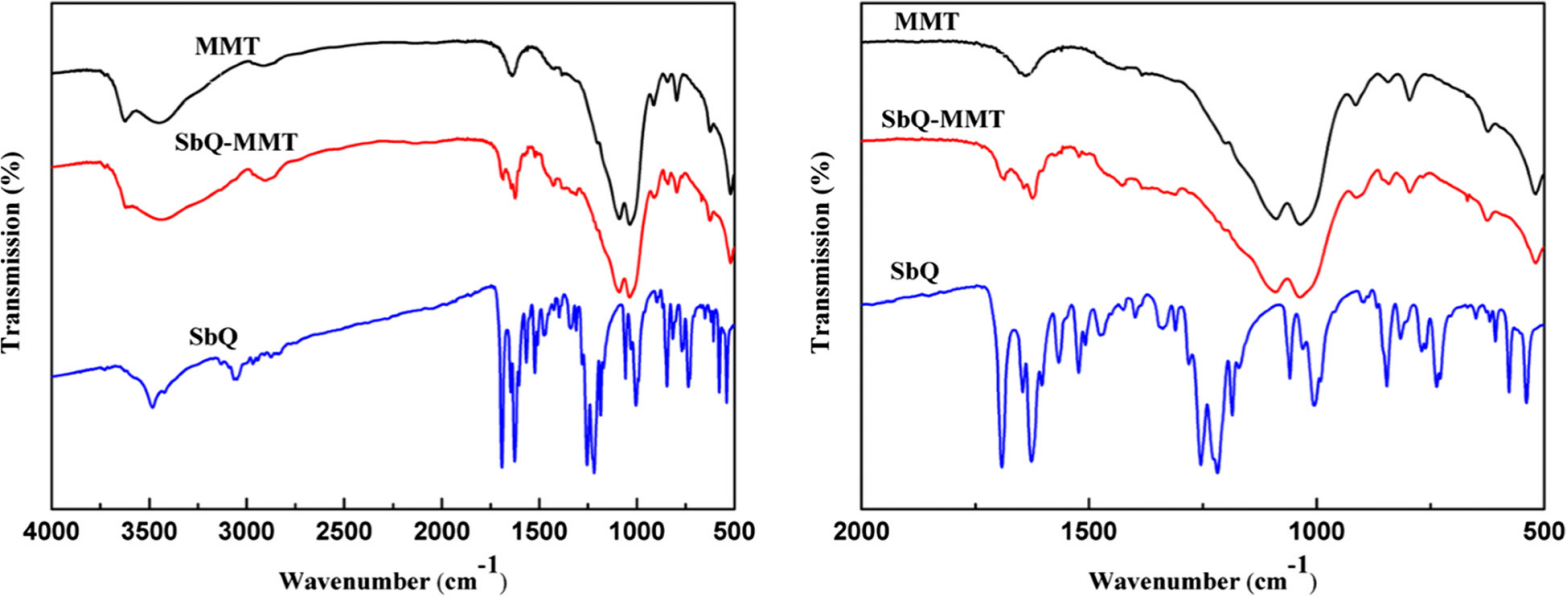
FTIR spectra of pristine MMT, SbQ, and cross-linked SbQ-MMT.

Figure 5 shows the FTIR spectra of MMT, SbQ, and cross-linked SbQ-MMT. The peaks exhibited at 3,435, 1,639, and 1,163 to 500 cm^−1^ were − OH stretching, −OH bending, and oxide bands of metals like Si, Al, and Mg. The shoulders and broadness of the structural − OH band were mainly due to contributions of several structural − OH groups, occurring in the MMT. The overlaid absorption peak at 1,640 cm^−1^ was attributed to − OH bending mode of adsorbed water. Peaks at 935, 850, and 825 cm^−1^ could be attributed to AlAlOH, AlFeOH, and AlMgOH bending vibrations, respectively [18]. In the FTIR spectrum of cross-linked SbQ-MMT, characteristic bands belonging to MMT and SbQ appeared, indicating that the cross-linked SbQ had interacted with MMT. The band which appeared at 1,650 cm^−1^ indicated the aldehydic (−CHO) group of SbQ which could interact with the − NH_2_ groups present in protein for drug delivery.

**Figure 6:**
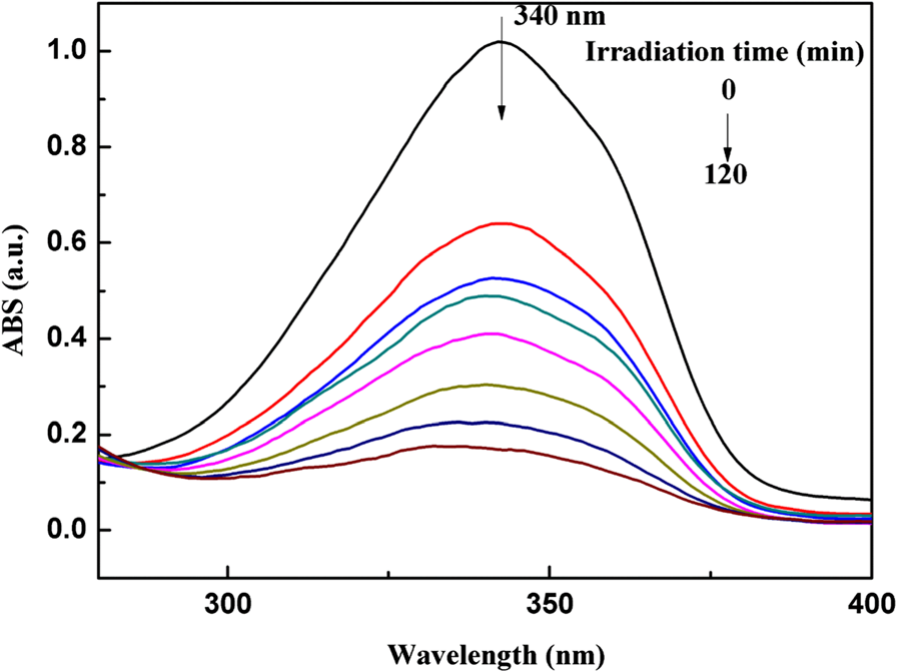
UV-vis spectra of the photo-cross-linking process of SbQ-MMT solution as a function of irradiation time.

UV-vis spectroscopy was utilized to trace the photo-cross-linking process of SbQ-MMT solution (Figure 6). When the solution was exposed to UV light, the absorbance intensity at around 340 nm decreased continuously with increased irradiation time, which indicated the dimerization of SbQ moieties [5]. SbQ moieties were completely cross-linked after 120 min.

## Conclusions

In summary, SbQ was successfully intercalated into MMT via cationic exchange interactions and were irradiated under UV light to get the cross-linked SbQ-MMT. The present study was the first to our knowledge to use SbQ to intercalate into MMT layers. The results showed that the layered basal spacing of MMT was increased and the morphology of MMT was changed after the intercalation of SbQ. It was found that SbQ was cross-linked after UV irradiation as designed. The existence of aldehyde (−CHO) group, the hydrophobic character of cross-linked SbQ molecules and the natural properties of MMT make these novel materials to be potentially used in drug delivery or as an additive into polymeric composites to improve their mechanical properties.

## Abbreviations

SbQ: a styrylpyridinium salt; MMT: montmorillonite; SEM: scanning electron microscopy; AFM: atomic force microscopy; XRD: X-ray diffraction; TEM: transmission electron microscopy; TGA: thermal gravimetric analysis; FTIR: Fourier transform infrared spectroscopy.

## Competing interests

The authors declare that they have no competing interests.

## Authors’ contributions

QW and QW gave the guidance, and JC did the experiments. QW, XC, and JC analyzed the data and gave the final approval of the version of the manuscript to be published. All authors read and approved the final manuscript.

## Authors’ information

JC, female, current master student, has a research direction of functional nanofibers. QW, male, professor, has a research field of functional nanofibers.
